# Antibody-induced pain-like behavior and bone erosion: links to subclinical inflammation, osteoclast activity, and acid-sensing ion channel 3–dependent sensitization

**DOI:** 10.1097/j.pain.0000000000002543

**Published:** 2021-11-19

**Authors:** Alexandra Jurczak, Lauriane Delay, Julie Barbier, Nils Simon, Emerson Krock, Katalin Sandor, Nilesh M. Agalave, Resti Rudjito, Gustaf Wigerblad, Katarzyna Rogóż, Arnaud Briat, Elisabeth Miot-Noirault, Arisai Martinez-Martinez, Dieter Brömme, Caroline Grönwall, Vivianne Malmström, Lars Klareskog, Spiro Khoury, Thierry Ferreira, Bonnie Labrum, Emmanuel Deval, Juan Miguel Jiménez-Andrade, Fabien Marchand, Camilla I. Svensson

**Affiliations:** a Department of Physiology and Pharmacology, Center for Molecular Medicine, Karolinska Institutet, Stockholm, Sweden; b Université Clermont Auvergne, Inserm U1107 Neuro-Dol, Pharmacologie Fondamentale et Clinique de la Douleur, Clermont-Ferrand, France; c Université Clermont Auvergne, Inserm UMR 1240, IMoST, Imagerie Moléculaire et Stratégies Théranostiques, Clermont-Ferrand, France; d Unidad Academica Multidisciplinaria Reynosa Aztlan, Universidad Autonoma de Tamaulipas, Reynosa, Tamaulipas, Mexico; e Department of Biochemistry and Molecular Biology, University of British Columbia, Vancouver, BC, Canada; f Department of Medicine, Division of Rheumatology, Center for Molecular Medicine, Karolinska University Hospital, Stockholm, Sweden; g Lipotoxicity and Channelopathies (LiTch)—ConicMeds, Université de Poitiers, Poitiers, France; h Université Côte d’Azur, CNRS, IPMC, LabEx ICST, FHU InovPain, France

**Keywords:** Pain, Osteoclasts, Bone, Autoantibodies, Rheumatoid arthritis, ASIC3, Lipids, LPC, sPLA_2_

## Abstract

Several bone conditions, eg, bone cancer, osteoporosis, and rheumatoid arthritis (RA), are associated with a risk of developing persistent pain. Increased osteoclast activity is often the hallmark of these bony pathologies and not only leads to bone remodeling but is also a source of pronociceptive factors that sensitize the bone-innervating nociceptors. Although historically bone loss in RA has been believed to be a consequence of inflammation, both bone erosion and pain can occur years before the symptom onset. Here, we have addressed the disconnection between inflammation, pain, and bone erosion by using a combination of 2 monoclonal antibodies isolated from B cells of patients with RA. We have found that mice injected with B02/B09 monoclonal antibodies (mAbs) developed a long-lasting mechanical hypersensitivity that was accompanied by bone erosion in the absence of joint edema or synovitis. Intriguingly, we have noted a lack of analgesic effect of naproxen and a moderate elevation of few inflammatory factors in the ankle joints suggesting that B02/B09-induced pain-like behavior does not depend on inflammatory processes. By contrast, we found that inhibiting osteoclast activity and acid-sensing ion channel 3 signaling prevented the development of B02/B09-mediated mechanical hypersensitivity. Moreover, we have identified secretory phospholipase A2 and lysophosphatidylcholine 16:0 as critical components of B02/B09-induced pain-like behavior and shown that treatment with a secretory phospholipase A2 inhibitor reversed B02/B09-induced mechanical hypersensitivity and bone erosion. Taken together, our study suggests a potential link between bone erosion and pain in a state of subclinical inflammation and offers a step forward in understanding the mechanisms of bone pain in diseases such as RA.

## Abstract 2

Supplemental Digital Content is Available in the Text.

Inhibiting osteoclast activity and acid-sensing ion channel 3 signaling prevents the development of autoantibody-mediated mechanical hypersensitivity, and increased lysophosphatidylcholine 16:0 and secretory phospholipase A2 contribute to sensitization in this model.

## 1. Introduction

Many different pathological bone conditions, including bone cancer, osteoporotic fractures, and rheumatoid arthritis (RA) are associated with a high risk of developing persistent pain.^7,41,63^ Preclinical data indicate that osteoclast-inhibiting drugs, such as bisphosphonates and antireceptor activator of nuclear factor-kappa B ligand (RANKL) antibodies, are antinociceptive in animal models of different bone pathologies.^20,64,66,88,104^ Moreover, in some human studies, these types of drugs are also associated with pain relief.^1,17,59^ Thus, increased osteoclast activity may lead not only to increased bone resorption and structural changes in bone microarchitecture but also to the production of algogenic factors that sensitize nociceptors innervating the bone.

Osteoclasts secrete protons (H^+^) through vacuolar H^+^-ATPase (V-ATPase) which, together with enzymes such as cathepsin K and matrix metalloproteinases, enable bone matrix degradation.^91^ Nociceptors respond to H^+^ mainly through acid-sensing ion channel 3 (ASIC3) and transient receptor potential vanilloid type 1 (TRPV1), and inhibition of these receptors attenuates pain-related behaviors in experimental models of bone pain.^25,29,31,36,83^ Osteoclasts can also produce other pronociceptive factors, including lipids,^51^ chemokines,^98^ and neurotrophic factors.^104^ Hence, osteoclasts may contribute to pain-like behaviors in various ways. Bone-related pain is often associated with tissue inflammation, neuronal sprouting, or tumor growth^57,68^ and in such conditions several pronociceptive mechanisms are likely acting in synchrony. Interestingly, induction of osteoclastogenesis by local or systemic administration of RANKL does not produce pain-related behaviors in mice, suggesting that enhanced osteoclast activity in the absence of other local changes is not pain-inducing.^16^ Thus, the exact role of osteoclasts and how they contribute to sensitization of nociceptors are not fully understood yet.

Historically, bone loss in RA has been considered a consequence of synovial inflammation, but recent reports suggest that bone degradation starts even before the onset of clinical symptoms.^47,60^ Joint pain is also an early indicator of emerging RA.^11,75^ Furthermore, many patients with established RA continue to report moderate to severe pain despite a marked reduction in inflammation and disease activity in response to antirheumatic treatment.^52^ This discrepancy between disease activity and pain indicates that synovitis is not the only driver of joint pain in RA^3,48^ and that additional mechanisms are at play. Indeed, pain-associated behaviors are present before and after the phase of joint inflammation observed in animals subjected to antibody-induced arthritis.^5,15,23^ In our previous work, we found that mice injected with osteoclast-activating monoclonal antibodies (mAbs) derived from synovial B cells from patients with RA displayed bone loss and pain-related behaviors without any visible signs of inflammation.^4,50,86,98^ Although the definitive targets of these antibodies are unknown, we have here used one of them, 1103:01B02 (B02), together with 1325:01B09 (B09), which preferentially binds to acetylated and citrullinated peptides.^55,81,86^ The combination of B02 and B09 mAbs was used as a tool to examine osteoclast contribution to pain-related behaviors that occur in the absence of overt tissue pathology. Thus, the aim of the current study was to examine the contribution of osteoclast activity to mechanical hypersensitivity induced by mAbs derived from patients with RA.

## 2. Methods

### 2.1. Mice

Because the prevalence of RA is higher in women than in men (3:1) we only used female mice in this study. BALB/c female mice (10-12 weeks old) were purchased from Janvier labs (Le Genest-Saint-Isle, France) or Charles River (Freiberg, Germany) and were housed in a temperature-controlled room with a 12-hour light–dark cycle, with food and water ad libitum. C57BL/6J WT-littermates and ASIC3 knock-out female mice^99^ were bred in the University of Clermont Auvergne Medical School animal facility (France) and housed as described above. For APETx2 pharmacological experiment, C57BL/6J female mice were purchased from Janvier labs. All procedures performed in France and Sweden were approved by the local ethical committees (Comité Régional d’éthique en matière d'expérimentation animale Auvergne and Stockholm Norra Djurförsöksetiska nämnd, respectively). All experiments were performed in accordance with the European Communities Council Directive for the care of laboratory animals (86/609/EEC) as well as with the International Association for the Study of Pain and ARRIVE guidelines. The number of animals used for each experiment is listed in the figure legends.

### 2.2. Monoclonal antibodies

Synovial fluid samples were drawn from knee joints of 2 female patients with RA based on the clinical need to alleviate the symptoms of active joint inflammation. Both patients attended the Rheumatology clinic at Karolinska University Hospital and fulfilled RA criteria as defined by the American College of Rheumatology 1987. Informed consent to use the samples for research was obtained from the patients in accordance with a protocol approved by the Ethical Review Committee North (KI forskningsetikkommitté Nord) of Karolinska University Hospital. Both patients were carrying Major Histocompatibility Complex, Class II, DR Alpha (HLA-DR) shared epitope allele and were seropositive, with high anticyclic citrullinated antibodies (CCP) levels measured by CCPlus (Euro Diagnostica, Malmö, Sweden). Monoclonal antibodies were generated from single memory B cells^54^ (1103:01B02 and control mAbs 1362:01E02 and 1276:01G09) or antibody-secreting cells (1325:01B09) isolated from synovial fluid mononuclear cells from the patients with RA.^86^ After isolation, Ig variable regions were cloned into vectors and mAbs were expressed as recombinant immunoglobulin G (IgG) in Expi293 human embryonic kidney (HEK) cells (Life Technologies, Carlsbad, CA). Antibodies were purified using Protein G Sepharose 4 Fast Flow resin (GE Healthcare, Stockholm, Sweden) and assessed for reactivity using a range of modified peptide and/or protein Enzyme-Linked Immunosorbent Assay (ELISA) and a custom-designed autoantigen microarray (Thermo Fisher Scientific—Immunodiagnostics, Uppsala, Sweden),^32^ as previously described.^81,86^ In-depth binding was also investigated with a large extracellular matrix peptide microarray with 53019 cit-peptides, 49022 carb-peptides, and equivalent native control peptides (Roche NimbleGen Pleasanton, CA, US). The exact steps in the process of cloning and expression have been described elsewhere.^4^ All IgGs were endotoxin tested (<10 EU/mg IgG) and extensively quality-controlled with ELISA, sodium dodecyl-sulfate polyacrylamide gel electrophoresis (SDS-PAGE), and size exclusion chromatography. Murine chimera antibodies of the human mAbs (hB02, hB09) were generated by replacing human gamma and lambda/kappa constant regions with the murine IgG2a constant regions. The 1325:01B09 antibody binds to citrullinated, acetylated, and carbamylated peptides and is classified as an antimodified protein antibody. The 1103:01B02 antibody is negative for reactivity towards all screened citrulline-containing and arginine-containing peptides. 1103:01B02 does not show polyreactivity or general nonspecific reactivity,^81^ despite it previously having had functional activity and osteoclast binding,^50^ it is currently not known which epitopes 1103:01B02 binds to. Two control antibodies, 1276:01G09 and 1362:01E02, have not had any detectable reactivity. Human and murine chimera mAbs were used interchangeably in this study because we have previously confirmed that both yield comparable effects on pain-like behavior and bone morphology. The mAb 1103:01B02 (human and murine) will be referred to as B02, the 1325:01B09 (human and murine) as B09, and the control mAbs 1362:01E02 and 1276:01G09 (human and murine) as E02 and G09, respectively. Each mAb was injected intravenously alone (2 mg) or in combination (B02/B09; 1 mg each).

### 2.3. Collagen antibody–induced arthritis

Collagen antibody–induced arthritis (CAIA) was induced as previously described.^5^ In brief, mice were injected i.v. with anticollagen type II arthritogenic cocktail (1.5 mg/mouse; Chondrex, Woodinville, WA) on day 0. Lipopolysaccharide (LPS, 25 μg/mouse, Chondrex) was injected intraperitoneally (i.p.) on day 5 to synchronize the onset of joint inflammation. The control group received saline solution i.v. on day 0 and i.p. on day 5. Mice were killed 16 days after CAIA induction at the peak of inflammation.

### 2.4. Arthritis scoring

The development of joint inflammation in the forepaws and hind paws was monitored daily through visual inspection, performed by a blinded researcher, as previously described.^67^ In brief, 1 point was given for each inflamed toe or knuckle, and if the ankle and/or wrist was inflamed, 5 points were given, resulting in a maximum arthritis score of 15 points per paw and 60 points per mouse.

### 2.5. Behavioral tests

Paw mechanical sensitivity was assessed using von Frey hairs based on the up–down method.^14^ von Frey hairs used in France for APETx2, ASIC3 KO, paracetamol and morphine experiments were as follows: 0.02, 0.04, 0.07, 0.16, 0.4, 0.6, 1.0, and 1.4 g (Bioseb, Vitrolles, France). In Sweden, von Frey OptiHair (Marstock OptiHair, Schriesheim, Germany) was used with a logarithmically incremental stiffness of 0.5, 1, 2, 4, 8, 16, and 32 mN (converted to 0.051, 0.102, 0.204, 0.408, 0.815, 1.63, and 3.26 g, respectively). A cutoff of 4 g was applied to avoid tissue damage, and thus, nociceptive responses in the suprathreshold range were not assessed. Animals were acclimatized for at least 1 hour in an individual clear acrylic cubicle placed on the top of an elevated wire mesh. Three baselines were measured on 3 different days followed by randomization of the mice into different groups. The filaments were applied on the right and left hind paws alternatively, with a 5-minute interval between stimuli. Quick withdrawal or licking and/or biting of the paw to the stimulus was considered a positive response. Withdrawal threshold (the threshold of mechanical hypersensitivity at which there was a 50% probability of paw withdrawal) was calculated for both hind paws and averaged. The 50% paw mechanical threshold was evaluated before and at different time points (refer to Pharmacological experiments section) after control antibody, phosphate-buffered saline (PBS), or B02/B09 administration. The investigator performing behavioral testing was blinded to the experimental groups and treatments throughout the whole study.

### 2.6. Quantitative real-time polymerase chain reaction

Ankle joints of the hind legs were processed for gene expression analysis 14 days post-B02/B09 injection and 16 days post-CAIA. Ankle joints were dissected out and trimmed 6 mm from each side of the joint capsule, which was followed by removal of muscles and tendons. Samples were flash frozen and stored in −70°C until further processing. Joint tissue was pulverized with BioPulveriser (BioSpec, Bartlesville, OK), followed by bead homogenization using TissueLyser II (Qiagen, Hilden, Germany) in TRIzol reagent (Invitrogen, Carlsbad, CA). Total RNA was extracted according to the manufacturer's protocol and followed by a reverse transcription reaction using the High-capacity cDNA Reverse Transcription Kit (Invitrogen). qPCR was performed using a StepOne Real-Time PCR System (Applied Biosystems, Foster City, CA) or BioRad CFX RT-PCR System (Bio-Rad Laboratories, Stockholm, Sweden) using hydrolysis probes to measure the relative mRNA levels. Predeveloped probes (Applied Biosystems) for *Acp5* (Mm00475698_m1), *Ctsk* (Mm00484039_m1), *Tcirg1* (Mm00469406_m1), *Asic3* (Mm00805460_m1), *Asic1* (Mm01306001_g1), *Il1β* (Mm00434228_m1), *Tnf* (Mm00443258_m1), *Il6* (Mm00446190_m1), *Cxcl1* (Mm04207460_m1), *Cxcl2* (Mm00436450_m1), *Ptgs2* (Mm00478374_m1), *Pla2g2a* (Mm00448160_m1), *Pla2g5* (Mm00448162_m1), *Pla2g10* (Mm01344436_g1), *Enpp2* (Mm00516572_m1), *Lpar1* (Mm00439145_m1), *Lpar2* (Mm00469562_m1), *Lpar3* (Mm00469694_m1), *Lpar4* (Mm01228532_m1), *Lpar5* (Mm01190818_m1), and *Lpar6* (Mm00163058) were used for mRNA analysis. Data were normalized against housekeeping genes: *Rplp2* (Mm00782638_s1) or *Hprt* (Mm03024075_m1). Relative fold changes were calculated by the comparative Ct method (2^−ΔΔCT^).

### 2.7. Immunohistochemistry

Mice were terminally anaesthetized using pentobarbital and quickly perfused transcardially with saline, followed by 4% paraformaldehyde (PFA). After perfusion, L3-L4-L5 left dorsal root ganglia (DRG) were excised, postfixed 4 hours in the same perfusion fixative, cryoprotected in 30% sucrose in 0.1 M phosphate buffer for 48 hours at 4°C, and frozen in optimal cutting temperature (OCT; Histolab, Göteborg, Sweden) compound. Transverse DRG sections (12 μm) were cut on a cryostat, dried for 30 minutes at room temperature, and stored at −20°C before staining.

Dorsal root ganglion sections were stained as follows: After 3 washes with PBS, sections were blocked with PBS containing 0.2% Triton X-100 and 1% bovine serum albumin (BSA), Sigma-Aldrich BSA to reduce nonspecific binding. Sections were then incubated overnight at room temperature in a humid chamber with primary antibody (Guinea pig anti-ASIC3, 1/200, #AB5927; Merck Millipore, Molsheim France) diluted in PBS + 0.2% Triton X-100 and 1% BSA. After 5 washes 5 minutes each with PBS, sections were incubated with the appropriate secondary antibody (goat anti-guinea pig Alexa 488; Alexa Fluor, Life Technology, Ilkirch, France) in PBS + 0.2% Triton X-100 + 1% BSA 2 hours at room temperature. Sections were then washed 3 times in PBS and cover-slipped with fluorescent mounting medium (Dako, Glostrup, Denmark). Negative control sections were stained only with the secondary antibody and subjected to the same experimental conditions. Sections were visualized under a Nikon Eclipse Ni-E fluorescent microscope run with Nikon analysis software (NiS element). Counting of ASIC3-positive DRG neurons in L3-L4-L5 DRG (4-6 sections for each animal, n = 5 per group) was performed by a blinded experimenter. Signal above background in the form of punctate staining in cytoplasm and/or membrane was considered as a positive cell.

### 2.8. Joint histology

Hind ankle joints were postfixed in 4% PFA (Histolab, Göteborg, Sweden) for 48 hours. Next, tissue was decalcified in 10% EDTA (Sigma-Aldrich, St Louis, MO) for 4 to 5 weeks, dehydrated in ethanol, and embedded in paraffin. Sagittal sections (5 μm) were cut and stained with hematoxylin and eosin (Histolab) and scored by 2 blinded investigators on a scale from 0 to 3, where 0 is normal and 3 is severe synovitis, bone erosion, and/or cartilage destruction, as previously described.^5^

### 2.9. Lipocalin-2 ELISA

At day 36 after B02/B09 or PBS administration, mice were terminally anaesthetized using pentobarbital (n = 8/group). The right ankle joint was excised and snap frozen in liquid nitrogen. Tissue samples were then crushed in liquid nitrogen with a pestle and mortar and subsequently homogenized in lysis buffer (137 mM NaCl, 20 mM TrisHCl, 1% NP40, 10% glycerol, 1 mM PMSF, 10 µg/mL aprotinin, 1 µg/mL leupeptin, and 0.5 mM sodium vanadate) using a glass homogenizer. Homogenates were then centrifuged at 14,000×*g* for 10 minutes at 4°C, and supernatants were collected. Whole-cell lysates were next titrated to determine their protein concentrations using Pierce BCA Protein Assay kit (Fisher Scientific, Loughborough, United Kingdom). Lipocalin-2 (LCN2) was measured using a quantitative sandwich enzyme immunoassay technique according to the manufacturer's guidelines (DuoSet, # DY1857; R&D system, Minneapolis, MN). Samples were processed in duplicates, and values were calculated from the standard curve.

### 2.10. Two-DeoxyGlucosone 750 imaging

Glucose uptake as a surrogate of tissue inflammation assessment was measured in vivo using an IVIS Spectrum small animal imaging system (Perkin Elmer) at day 18 after B02/B09 or PBS administration (n = 8 in each group). Each mouse received 10 nmol/100 µL of XenoLight RediJect 2-deoxyglucosone (DG)-750 (Perkin Elmer) by i.v. administration. Three hours later, acquisition was performed under isoflurane anesthesia (1.5%). Imaging was performed using the ex/em = 745/820 nm bandpass filters. Quantification analysis was performed with Living Image Software using region of interest (ROI) delineated on hind paws (ankle joint). The results are presented as the average of both hind paws of total counts of radiance efficiency (radiance [photons per second per square centimeter per steradian] per incident excitation power [microwatt per square cm]).

### 2.11. Microcomputed tomography

At day 28, animals from each group (saline, 1276:01G09, 1103:01B02, 1325:01B09, and 1103:01B02+1325:01B09; n= 6-19 per group) were terminally anaesthetized using isoflurane or pentobarbital and quickly perfused transcardially with saline followed by 4% PFA. The right leg was dissected out, skin and muscles were carefully removed to avoid any bone damage, and postfixed in the same perfusion fixative for 48 hour. After 3 washes with PBS, the right leg was stored in 0.01M PBS at 4°C until scanning.

The trabecular bone was analyzed at the level of proximal tibia and talus using a microcomputed tomography (micro-CT) system (Skyscan 1272; Bruker, Kontich, Belgium). The scanning process was performed according to the guidelines for micro-CT analysis for rodent bone structure. The scanning was made at a 10-µm voxel size with an X-ray power of 60 kVp and 166 µA with an integration time of 627 ms. Obtained images were reconstructed using NRecon software (Bruker). The trabecular ROI was evaluated by selecting 2 mm in the vertical axis, subsequent to 0.5 mm from the growth plate (reference point). The CT analyzer program (Bruker) was used to determine trabecular bone parameters; an automatic segmentation algorithm (CT analyzer) was applied to isolate the trabecular bone from the cortical bone. The parameters used for the trabecular bone were bone mineral density, trabecular bone volume ratio (BV/TV), trabecular separation (Tb.Sp), and trabecular number (Tb.N). Finally for cortical bone, cortical thickness (Ct.Th) was obtained.

### 2.12. Cathepsin K activity imaging

Bone resorption was assessed in vivo using a small animal IVIS Spectrum imaging system (Perkin Elmer, Waltham, MA) using the CatK680 FAST fluorescent imaging agent (Perkin Elmer), which localizes to sites of increased bone resorption and osteoclast activity.^82^ On day 21 after B02/B09 or PBS administration, 2 nmol/100 µL Cat K 680 FAST (Perkin Elmer) was injected i.v. (n = 5/group) and image acquisition was performed 6 hours later under isoflurane anesthesia (1.5%) using the ex/em = 675/720 nm bandpass filters. Quantification analysis was performed as described for 2-DG fluorescent imaging.

### 2.13. Scintigraphic imaging

To evaluate cartilage remodeling, in vivo scintigraphic imaging was performed in the same animals with ^99m^Technetium-N-[triethylammonium]-3-propyl-^15^ ane-N5 (NTP 15-5) at days 2 and 28 after B02/B09 or PBS administration (n = 8-11 for each group). Planar acquisition (10 minutes, 15% window at 140 keV) was performed 30 minutes after i.v. administration of 25MBq of ^99m^Tc-NTP 15-5 on the animal placed in ventral decubitus on a parallel collimator (20 mm/1.8/0.2) of a gamma camera for small animals (γImager, Biospace, Nesles-la-Vallée, France) under isoflurane anesthesia (1%). To evaluate bone remodeling, in vivo scintigraphic imaging was also performed with ^99m^Technetium-hydroxymethylene diphosphonate (^99m^Tc-HMDP, Osteocys, IBA) radiotracer at days 4 and 31 after B02/B09 or PBS administration (n=8-11 for each group). The bone imaging phase was acquired 2.5 hours after intravenous injection of 10 MBq per mouse of ^99m^Tc-HMDP. Planar acquisition was performed (5 minutes, 15% window at 140 keV) as described for ^99m^Tc-NTP 15-5. Quantitative analysis of both scintigrams was performed with *GammaVision+*, with ROIs delineated over the hind paws, and results are presented as the average of both hind paws of the total counts in cpm in the ROI. For each radiotracer, the injected dose to each animal was determined by acquiring an image of the syringe before and after injection and counting using an activimeter (Capintec, Florham Park, NJ).

### 2.14. Pharmacological experiments

Ten days after B02/B09 injection, when mechanical hypersensitivity has developed, animals received subcutaneous (s.c.) injections of naproxen (50 mg/kg, Sigma-Aldrich) or saline during 3 consecutive days (days 10, 11, and 12; n=6-7 per group). Mechanical withdrawal thresholds were assessed 180 minutes after each injection. To reduce the number of animals used for this study, we allowed 6 days of drug washout, after which mice were subjected to s.c. injections with a secretory phospholipase A2 (sPLA_2_) inhibitor (varespladib, 3 mg/kg for 3 days followed by 6 mg/kg for 6 days, BioNordika, Stockholm, Sweden) or vehicle (20% DMSO in PBS) twice daily on days 19 to 24 post-B02/B09 injection (n=6-7 per group).

In the second cohort of mice, 14 days after B02/B09 or PBS injection, mice received either saline (s.c.) or morphine (3 mg/kg, s.c. Tocris, Bristol, United Kingdom), just after baseline threshold measurement (n = 8 per group). Mechanical thresholds were assessed 30 and 60 minutes posttreatment. After 1 week of drug washout (ie, 21 days after B02/B09 or PBS injection), mice received either saline (s.c.) for the one previously treated with morphine or paracetamol (200 mg/kg, Sigma-Aldrich) for the one previously treated with saline, just after baseline threshold measurement. Mechanical thresholds were then assessed at 45 minutes postinjection.

In the third cohort of mice, 2 different osteoclast inhibitors, bisphosphonate (zoledronate, Sigma-Aldrich) and cathepsin K inhibitor tanshinone IIA sulfonic sodium (T06, kindly provided by Dr. Dieter Brömme, University of British Columbia, Vancouver, Canada), were used.^70^ T06 (40 mg/kg) was resuspended in distilled water and given by oral gavage every day for the whole duration of the study (1-14 days post-B02/B09 injection) in 100 μL/10g body weight volume. Zoledronate (100 µg/kg) or saline were injected intraperitoneally (i.p.) into mice on days 1, 4, 7, and 10 post-B02/B09 injection.

In the fourth cohort of mice, APETx2 (Alomone Labs, Jerusalem, Israel), a selective ASIC3 channel blocker,^19^ was injected at days 14 and 23 post-B02/B09 or PBS administration. Each mouse was injected intraarticularly (i.a.) into the right ankle joint (ipsilateral) with 20 µmol of APETx2, diluted in 10 µL of saline, under 1.5 to 2% isoflurane anesthesia. Mechanical thresholds were assessed before and after B02/B09 or PBS administration and 1, 4, and 24 hours after i.a. injection. The contralateral paw, injected with saline, was used as an internal control (n = 8 per group).

In the fifth cohort of mice, bafilomycin A1 (BafA1, Abcam, Cambridge, United Kingdom), a selective V-ATPase inhibitor was used. After the induction of mechanical hypersensitivity with B02/B09, mice (n = 6 per group) received daily s.c. injections of BafA1 (25 µg/kg) or vehicle (0.01% DMSO in PBS) for 5 consecutive days (days 12-16) and were tested for mechanical thresholds at 1 and 3 days posttreatment (days 17 and 19). Mice injected with 1362:01E02 (n = 3) were used as control.

### 2.15. Primary osteoclast cultures

Osteoclasts were derived from mononuclear hematopoietic cell population from the bone marrow of BALB/c female mice (16-20 weeks old). Hind limbs were carefully dissected keeping the long bones intact, followed by skin and muscle removal. Total bone marrow was flushed out from both tibia and femur using 1 mL-syringe filled with cold αMEM complete serum (Life Technologies, Carlsbad, CA). To remove cell aggregates, the cell suspension was pipetted up and down and flushed through a 70-μm cell strainer, followed by centrifugation for 7 minutes at 300g. Cells were resuspended in 15 mL of selection medium (αMEM) supplemented with 1:100 PEST (ThermoFisher) and 10% FBS (Sigma-Aldrich) and plated onto a T75 flask with a ventilated cap (Sarstedt, Nümbrecht, Germany). Cells were incubated at 37°C and 5% CO_2_ overnight to allow adherence of stromal cells. Next day, nonadherent cells were removed and harvested by centrifugation for 7 minutes at 300g, followed by counting using 0.4% Trypan blue stain (ThermoFisher). Cells were resuspended in αMEM supplemented with 30 ng/mL M-CSF and 50 ng/mL RANKL (R&D Systems, Minneapolis, MN) and seeded onto 96-well plates at a density of 1.00 × 10^6^ cells/mL and cultured for 5 days at 37°C and 7% CO_2_. Antibodies 1103:01B02, 1325:01B09, and 1276:01G09 were added to the culture at a concentration of 5 µg/mL on the day of seeding and on day 4, during media change. At the end of experiment, cells were fixed with 4% PFA, washed with 0.01M PBS, and tartrate-resistant acid phosphatase stained using a leukocyte acid phosphatase kit (Sigma-Aldrich). The number of multinucleated cells (1-3, 3-9, and >9nuclei) was counted and averaged for each condition (5 wells/condition). Observations have been repeated in 3 independent experiments.

### 2.16. Lipid extraction

Lysophosphatidylcholine (LPC) species content was assessed in bone marrow extracted from B02/B09-injected and PBS-injected groups 28 days postinjection. The lipid extraction was adapted from the Folch method.^24^ In brief, 50 μL of bone marrow sample was diluted in 950 μL of water and transferred into glass tubes with PTFE caps (PyrexLabware, Stoke-on-Trent, United Kingdom) containing 500 μL of glass beads (Ø 0.3-0.4 mm, Sigma-Aldrich). Mixtures were supplemented with 4 mL of a 2/1 (vol/vol) chloroform (CHCl_3_)/methanol (CH_3_OH) solution containing lipid standards (LPC17:0 or LPC13:0), and lipid extractions were performed on an orbital shaker (IKA VXR basic Vibrax; Sigma-Aldrich) at 1500 rpm for 2 hours at room temperature. From the resulting emulsions, aqueous phases were dissociated from the lipid-containing organic phases with a swing-out centrifuge for 5 minutes at 410 g. Aqueous phases were discarded, and organic ones were supplemented with 1 mL of a 4/1 (vol/vol) 2N KCl/CH_3_OH solution. Samples were shaken for 10 minutes, and organic and aqueous phases were separated as described before. The resulting organic phases were complemented with 1 mL of 3/48/47 (vol/vol/vol) CHCl_3_/CH_3_OH/H_2_O solution, and an additional shaking step was performed. The resulting organic phases were isolated, transferred into new glass tubes, and supplemented with 1 mL of a 3/48/47 (vol/vol/vol) CHCl_3_/CH_3_OH/H_2_O solution and shaken for 10 minutes. The final organic phases containing the whole lipids were isolated, solvent evaporated at 80°C under a nitrogen beam (1 bar), and stored at −80°C until analysis.

### 2.17. Phospholipid purification and mass spectrometry analysis

Purification of phospholipids from the total lipid extract was performed on a silica column (Bond ELUT-SI; Agilent Technologies, Santa Clara, CA) using 2 mL of a 50/45/5 (vol/vol/vol) CHCl_3_/CH_3_OH/H_2_O solution, after elution of nonpolar lipids and glycolipids with appropriate solutions. The eluted fractions containing the phospholipids were analyzed by direct infusion on a SYNAPT G2 High-Definition Mass Spectrometer HDMS (Waters Corporation, Milford, MA) equipped with an electrospray ionization source in the positive ion mode, as described elsewhere.^44^ All spectra were recorded with the MassLynx software (Version 4.1, Waters).

### 2.18. Electrophysiology

HEK293 cell lines were grown as described previously.^22^ One day after plating, cells were transfected with pIRES2-mASIC3-EGFP vectors using the JetPEI reagent according to the supplier's protocol (Polyplus transfection SA, Illkirch, France). Fluorescent cells were used for patch-clamp recordings 2 to 4 days after transfection. Whole-cell configuration of the patch-clamp technique was used to measure membrane currents (voltage clamp). Recordings were made at room temperature using an Axopatch 200B amplifier (Axon Instruments, Union City, CA) with a 2 kHz low-pass filter. Data were sampled at 10 kHz, digitized by a Digidata 1440 A-D/D-A converter (Axon Instruments and recorded on a hard disk using pClamp software (version 10; Axon Instruments). The patch pipettes (2-8 MΩ, borosilicate glass, WPI, Sarasota, FL) contained (in mM) the following: 135 KCl, 5 NaCl, 2 MgCl_2_, 5 EGTA, and 10 HEPES (pH 7.25 with KOH). The control bath solution contained (in mM) the following: 145 NaCl, 5 KCl, 2 MgCl_2_, 2 CaCl_2_, 10 HEPES, and 10 glucose (pH 7.4 with N-methyl-D-glucamine). ASIC currents were induced by shifting 1 of 8 outlets of the microperfusion system from a holding control solution (ie, pH 7.4) to an acidic test solution (ie, pH 7.0). Whole-cell recording experiments were performed at −80 mV, and acute application of B02 and B09 at 1 µg/mL or LPC 16:0 (Avanti Polar Lipids, Coger, France) at 10 µM was made at resting pH 7.4.

### 2.19. Quantification and statistical analysis

Statistical tests were performed using GraphPad Prism 8 (San Diego, CA). To compare differences between groups split into 2 independent variables, 2-way analysis of variance was used followed by the Bonferroni post hoc test. To analyze differences between 2 groups, unpaired 2-tailed student t-test was used, whereas comparisons of 3 groups or more were assessed by 1-way analysis of variance, followed by the Bonferroni post hoc test. All data are presented as mean ± SEM, and *P* values less than 0.05 were considered statistically significant.

## 3. Results

### 3.1. Monoclonal antibodies from patients with rheumatoid arthritis differ in their pronociceptive properties but can act in synergy to prolong pain-like behavior when combined

**Figure 1.**
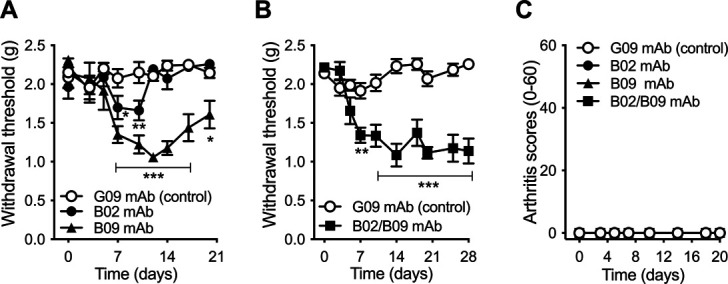
Monoclonal antibodies from patients with RA differ in their pronociceptive properties but can act in synergy to prolong pain-like behavior when combined. (A) A single i.v. injection of human B02 or B09 (2 mg/mouse) induces transient mechanical hypersensitivity when compared with human control antibody G09 (2 mg/mouse). (B) When B02 and B09 (1 mg each/mouse) are combined, the mAbs induce long-lasting pain-like behavior (28 days), compared with the control antibody (2 mg/mouse). (C) None of the human mAbs injected alone or in combination cause visual signs of inflammation (0-60). Data presented as mean±SEM, *P < 0.05, **P < 0.01, ***P < 0.001 compared with a control mAb; n = 6 to 11/group. mAb, monoclonal antibody; RA, rheumatoid arthritis.

To allow a side-by-side comparison, 2 different RA single-cell–derived recombinant human mAbs, B02 and B09, as well as human control antibody (G09), were injected alone (1 mg) or in combination (2 mg total, 1 mg/antibody) intravenously (i.v.) into female mice. Mice were then monitored for arthritis clinical scores and mechanical hypersensitivity. Although the G09 control antibody did not change the mechanical threshold throughout the whole experiment, the B09 mAb injected alone significantly reduced withdrawal thresholds from days 7 to 20 postinjection (Fig. 1A). By contrast, the B02 mAb only induced a transient mechanical hypersensitivity at days 7 and 10 postinjection. Interestingly, the administration of B02/B09 combination significantly increased and prolonged mechanical hypersensitivity measured from days 7 up to 28 postinjection, compared with B09 and B02 alone (Fig. 1B). This result suggests a potentiation of the pronociceptive effect of B09 when combined with B02. None of the mAbs injected alone or in combination induced any visual signs of joint inflammation (eg, edema or redness) throughout the study (Fig. 1C).

### 3.2. B02/B09-induced hypersensitivity is associated with subclinical inflammation

**Figure 2.**
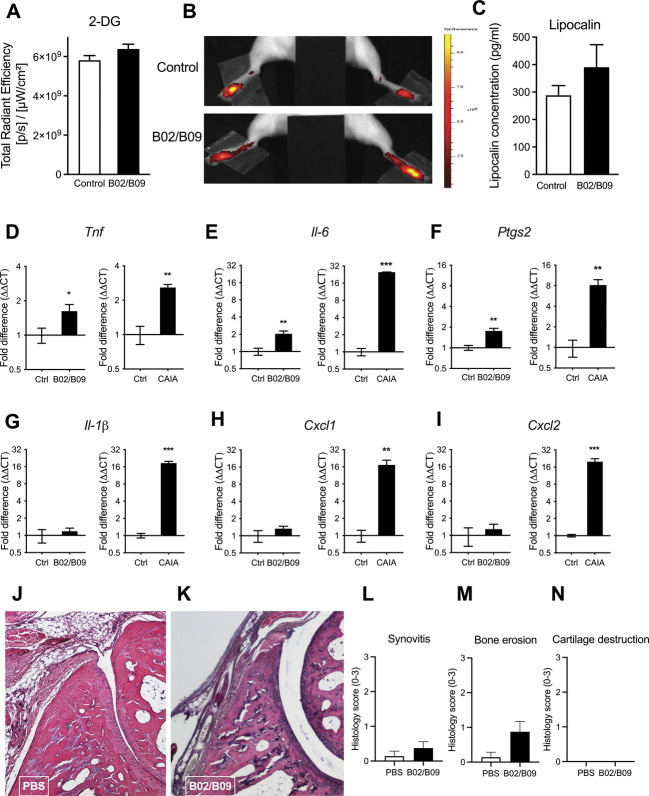
B02/B09-induced pain-like behavior is associated with subclinical inflammation. (A-B) B02/B09-injected mice do not show increased metabolic activity in the ankle joints, measured in vivo 3 hours after i.v. injection of 2-DG-750 luminescent probe (10 nmol/100 μL), day 14 post-mAb injection (n = 8-11/group, results are presented as total counts of radiant efficiency/incident excitation power and compared with the PBS-injected control group). (C) Lipocalin-2 levels measured in ankle joint homogenates 36 days post-mAb injection are not significantly elevated in the B02/B09 group compared with control (n = 8/group). Gene expression of (D) *Tnf*, (E) *Il-6*, and (F) *Ptgs2,* but not (G) *Il-1β*, (H) *Cxcl1,* and (I) *Cxcl2,* is significantly increased in ankle joints 14 days post-B02/B09 injection compared with the vehicle-injected control group (PBS), whereas mRNA for all factors is elevated in joints collected in the inflammatory phase of the CAIA model (day 16) (D–I), compared with the control group. The mRNA data were normalized to *Rplp2* mRNA levels and are presented as fold changes (n=5-6/group). (J–K**)** Representative ankle joint sections stained with hematoxylin and eosin 14 days after B02/B09 or PBS i.v. injection. (L–N) B02/B09 joints show signs of bone erosion but not synovitis or cartilage damage, compared with control (n = 7-8). Data presented as mean ± SEM, *P < 0.05, **P < 0.01, ***P < 0.001. 2-DG, 2-deoxyglucosone, CAIA, collagen antibody–induced arthritis; Ctrl, control.

To further investigate whether pain-related behavior induced by B02/B09 was driven by low-grade, subclinical joint inflammation, which cannot be detected by means of visual inspection, we performed 4 different types of analysis in ankle joints from mice injected with B02/B09 or PBS. In vivo assessment of changes in 2-DG fluorescently labelled glucose uptake, used as an indicator of inflammation, did not show any significant increase of 2-DG joint accumulation in the ankle joints 18 days after B02/B09 administration compared with the control group (Figs. 2A and B). Lipocalin-2, a protein released by neutrophils that regulates immune cell recruitment to the site of inflammation, was used as a marker of low-grade inflammation. It did not significantly increase in the ankle joint of the B02/B09 group compared with the control group at day 36 (Fig. 2C). Next, mRNA levels for a series of inflammation-associated factors were assessed in the ankle joints of B02/B09-injected and PBS-injected mice, 14 days post injection. Ankle joints from mice during the inflammatory phase of CAIA (day 16) were used as positive control. We found a significant but small increase of *Tnf* (Fig. 2D), *Il-6* (Fig. 2E), and *Ptgs2* (Fig. 2F) mRNA levels in the B02/B09 ankle joints but no significant changes in *Il-1β* (Fig. 2G), *Cxcl1* (Fig. 2H), or *Cxcl2* (Fig. 2I) mRNA levels compared with control joints. By contrast, mRNA levels for all 6 factors were elevated in ankle joints from CAIA mice compared with controls and with a much higher magnitude compared with B02/B09 joints. Finally, histological examination of ankle joints 14 days after B02/B09 or PBS injections (Figs. 2J and K) did not show signs of cell infiltration or synovial hyperplasia (Figs. 2L–N). These data suggest that B02/B09-induced mechanical hypersensitivity is accompanied by low-grade subclinical joint inflammation that cannot yet be detected by means of visual inspection or in vivo imaging.

### 3.3. B02/B09-induced mechanical hypersensitivity is reversed by morphine but not an nonsteroidal anti-inflammatory drug or paracetamol

**Figure 3.**
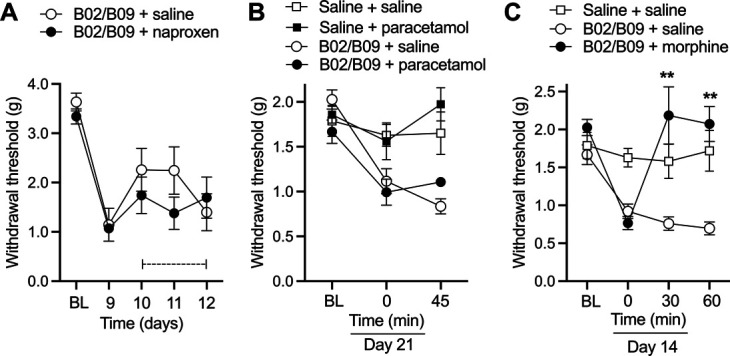
B02/B09-induced mechanical hypersensitivity is reversed by morphine but not an NSAID or paracetamol. (A) Repeated treatment with naproxen (50 mg/kg, i.p.) on days 10, 11, and 12 post-mAb injection does not reverse mechanical hypersensitivity induced by human B02/B09 antibodies. (B) Paracetamol (200 mg/kg; i.p.) administered on day 21 post-mAb injection has no analgesic effect in B02/B09-injected mice, measured 45 minutes post-drug administration. (C) Bolus injection of morphine (3 mg/kg, s.c.) administered on day 17 post-mAb reverses mechanical hypersensitivity induced by B02/B09, assessed 30 and 60 minutes post-drug administration. **P < 0.01, compared with saline vehicle control. Data presented as mean±SEM, n = 6 to 7/group. **P < 0.01, compared with saline vehicle control. Dashed line indicates dosing period. BL, baseline; NSAID, nonsteroidal anti-inflammatory drug.

Inflammation-induced pain is typically reduced by nonsteroidal anti-inflammatory drugs and paracetamol. To assess the contribution of inflammation to B02/B09-induced reduction in withdrawal thresholds, naproxen (50 mg/kg) was administrated once a day for 3 days. No reversal of mechanical hypersensitivity was observed compared with vehicle (Fig. 3A). Furthermore, paracetamol injection (200 mg/kg) did not reduce mechanical hypersensitivity in B02/B09-injected animals, compared with vehicle control. By contrast, systemic administration of 3 mg/kg morphine rapidly reversed mechanical allodynia at 30 and 60 minutes after injection compared with vehicle control. Taken together, these results indicate that B02/B09 antibody–induced mechanical hypersensitivity cannot be classified as a classical model of inflammatory pain but as a model in which pain-related behaviors are mainly driven by other mechanisms (Fig. 3B and C).

### 3.4. B02/B09 monoclonal antibodies alter bone homeostasis and induce bone erosion

**Figure 4.**
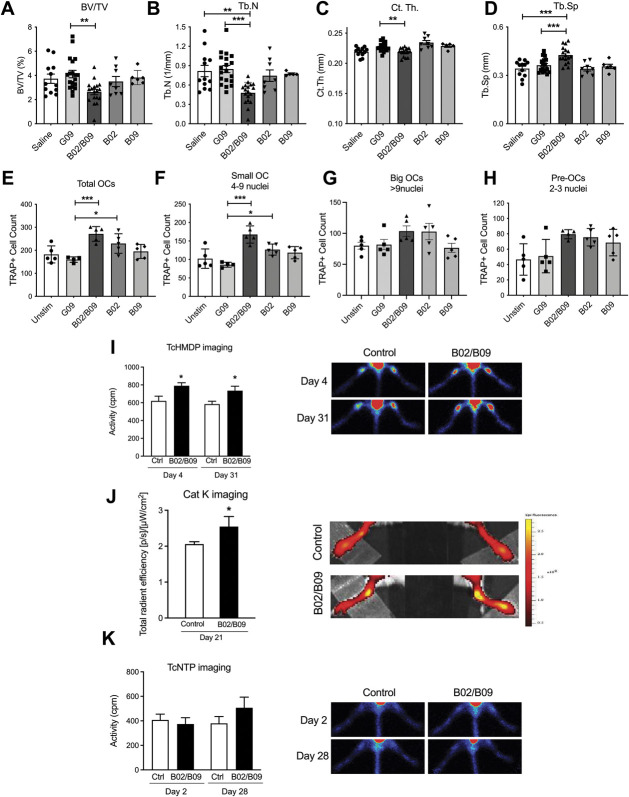
Intravenous injection of B02/B09 mAbs alters bone homeostasis and induces bone erosion. Micro-CT analysis of the proximal tibia shows that B02/B09 (2 mg i.v.) significantly reduces (A) trabecular bone volume (BV/TV), (B) trabecular number (Tb.N), and (C) cortical thickness (Ct. Th) and increases (D) trabecular separations (Tb. Sp) compared with G09 control (G09) or saline (n = 6-19/group). (E) Human B02/B09 increases the total number of TRAP+ osteoclasts in vitro with a significant effect on the number of (F) small osteoclasts (4-9 nuclei), but not (G) big osteoclasts (4-9 nuclei) or (H) preosteoclasts (2-3 nuclei), compared with a control antibody (G09) (n = 5 samples/group). (I) *In vivo* scintigraphy at days 4 and 31 using the ^99m^Tc-HMDP radiotracer (n = 8-12 mice/group) and (J) in vivo imaging assessing cathepsin K activity at day 21 show that B02/B09 injection stimulates bone remodeling (n = 5/group), compared with saline-injected control. (K) In vivo scintigraphy at days 2 and 28 using the ^99m^Tc-NTP 15-5 radiotracer shows that B02/B09 does not induce cartilage remodeling (n = 12-14/group), compared with saline-injected control. Data presented as mean±SEM, * *P* < 0.05, **P < 0.01, ***P < 0.001. Ctrl, control; OC, osteoclast; TcHMDP, technetium hydroxymethylene diphosphonate; TcNTP, technetium- N-[triethylammonium]-3-propyl-^15^ane-N5; Unstim, unstimulated.

We next tested whether B02 and B09, alone or in combination, have an impact on bone microarchitecture and osteoclast number. Micro-CT was used to evaluate alterations of bone density and microarchitecture in the proximal tibias from saline-injected, G09-injected, B02-injected, B09-injected, and B02/B09-injected mice at day 28 post injection (Figs. 4A–D). The morphometric parameters examined were bone volume fraction (BV/TV), Tb.N, Tb.Sp, and Ct.Th.. Although injecting the mAbs alone did not alter any of the parameters, the B02/B09 combination significantly reduced BV/TV, Tb.N, and Ct.Th and increased Tb.Sp compared with the control groups. There was no difference in morphometric parameters between mice injected with saline and the control mAb G09.

To assess the direct effect of the mAbs on osteoclast formation, we tested the effect of B02, B09, and the combination on bone marrow–derived osteoclast precursor cells in vitro. When combined, B02/B09 stimulated the formation of TRAP^+^ multinucleated cells (Fig. 4E), with a significant effect on the generation of small osteoclasts (4-9 nuclei; Fig. 4F), but none on the formation of big cells (>9 nuclei, Fig. 4G) or preosteoclasts (2-3 nuclei, Fig. 4H). Importantly, B02 alone also enhanced osteoclast formation, which is consistent with the earlier report on the B02 mAb effects on human osteoclasts.^50^

Next, to assess the temporal changes in B02/B09-driven bone metabolism, as well as the potential effect on cartilage remodeling, we performed scintigraphic and near-infrared fluorescent in vivo imaging. The ^99m^Tc-HMDP radiotracer is used for bone scanning because it accumulates in areas of active bone metabolism. We found a significant increased accumulation of ^99m^Tc-HMDP at days 4 and 31 after B02/B09 administration (Fig. 4I) compared with PBS administration. This indicates that the B02/B09 mAbs rapidly increased bone metabolism. Cat K 680 FAST is an activatable fluorescent agent that is optically silent on injection and produces fluorescent signal after cleavage by cathepsin K. A significant increase in cathepsin K signal in the ankle joint at day 21 after B02/B09 administration was observed compared with the PBS-injected group (Fig. 4J), reinforcing the micro-CT and ^99m^Tc-HMDP scintigraphic observations. ^99m^Tc-NTP targets proteoglycans and is used as a radioligand for in vivo functional monitoring of cartilage involvement.^62^ We did not detect any statistically significant increase of ^99m^Tc-NTP accumulation within the ankle joints at days 2 and 28 in mice injected with B02/B09 (Fig. 4K) compared with PBS injection, indicating a lack of in vivo cartilage remodeling. Overall, these results show that only B02 combined with B09 drive osteoclastogenesis in vitro and bone remodeling that leads to increased bone erosion in vivo.

### 3.5. Osteoclast inhibitors reverse B02/B09-induced mechanical hypersensitivity

**Figure 5.**
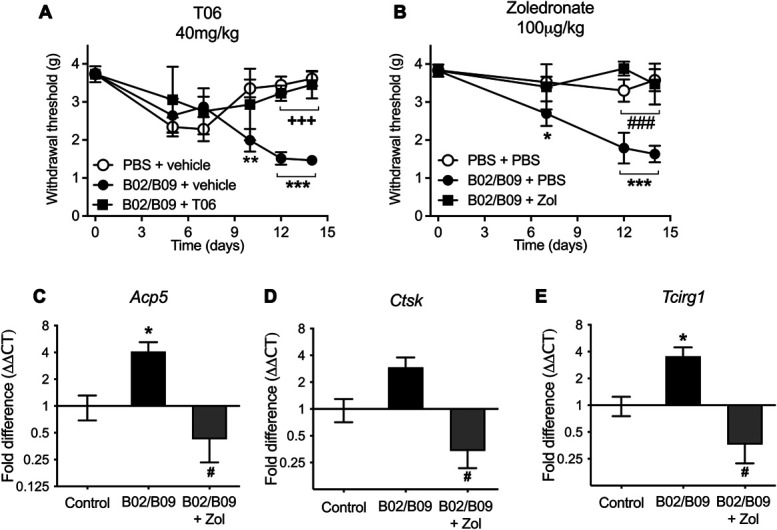
B02/B09-induced pain-like behavior is reversed by osteoclast inhibitors. (A) T06 (40 mg/kg) given daily by oral gavage for the whole duration of the study (1-14 days) prevents B02/B09-induced mechanical hypersensitivity, compared with the B02/B09 group treated with distilled water vehicle (n = 6-8/group). (B) Zoledronate (100ug/kg) administered i.p. on days 1, 4, 7, and 10 post-mAb injection prevents the development of B02/B09-induced pain-like behavior (n = 6/group). Statistical significance (2-way analysis of variance (ANOVA)) between B02/B09+vehicle and PBS+PBS/veh is marked by * and difference between B02/B09+vehicle and B02/B09+T06 or B02/B09+Zol is marked respectively with + or #. Data presented as mean±SEM; *P < 0.05; **P < 0.01; ***,###, or +++ *P* < 0.001. Increased gene expression of (C) *Acp5,* (D) *Ctsk*, and (E) *Tcirg1* in ankle joints of B02/B09-injected mice is prevented in zoledronate-treated mice (n = 6-12/group). Statistical significance (1-way ANOVA) between B02/B09-injected and PBS-injected control is marked by * and difference between B02/B09 and B02/B09+Zol is marked by #; * or #*P* < 0.05. T06, tanshinone IIA sulfonic sodium; Zol, zoledronate.

To assess whether the observed bone remodeling is involved in B02/B09-induced mechanical hypersensitivity, we examined the antinociceptive effect of 2 osteoclast inhibitors with different mechanisms of action. Daily administration of T06 (40 mg/kg), a cathepsin K inhibitor which inhibits osteoclast activity, prevented mechanical allodynia development after B02/B09 administration compared with the vehicle control group (Fig. 5A). In a separate experiment, mice received zoledronate (100 µg/kg), which is a nitrogen-containing bisphosphonate that leads to osteoclast apoptosis and prevents bone mineral dissolution and matrix digestion.^21^ When injected on days 1, 4, 7, and 10 after B02/B09 injection, zoledronate also prevented the development of mechanical allodynia in the B02/B09 group compared with the PBS control group (Fig. 5B). At the end of the zoledronate study, ankle joints were collected and processed for mRNA analysis by qPCR. A significant increase in mRNA levels of the osteoclast activity–associated factors, *Acp5* (Fig. 5C), *Ctsk* (Fig. 5D), and *Tcirg1* (Fig. 5E), were observed in the ankle joints of the B02/B9 group, 14 days post injection. Importantly, treatment with zoledronate completely abolished B02/B09-induced upregulation of these factors (Figs. 5C–E). These findings point towards a relationship between increased osteoclast activity and mechanical hypersensitivity in B02/B09-injected mice.

### 3.6. B02/B09-induced mechanical hypersensitivity is dependent on acid-sensing ion channel 3 activation

**Figure 6.**
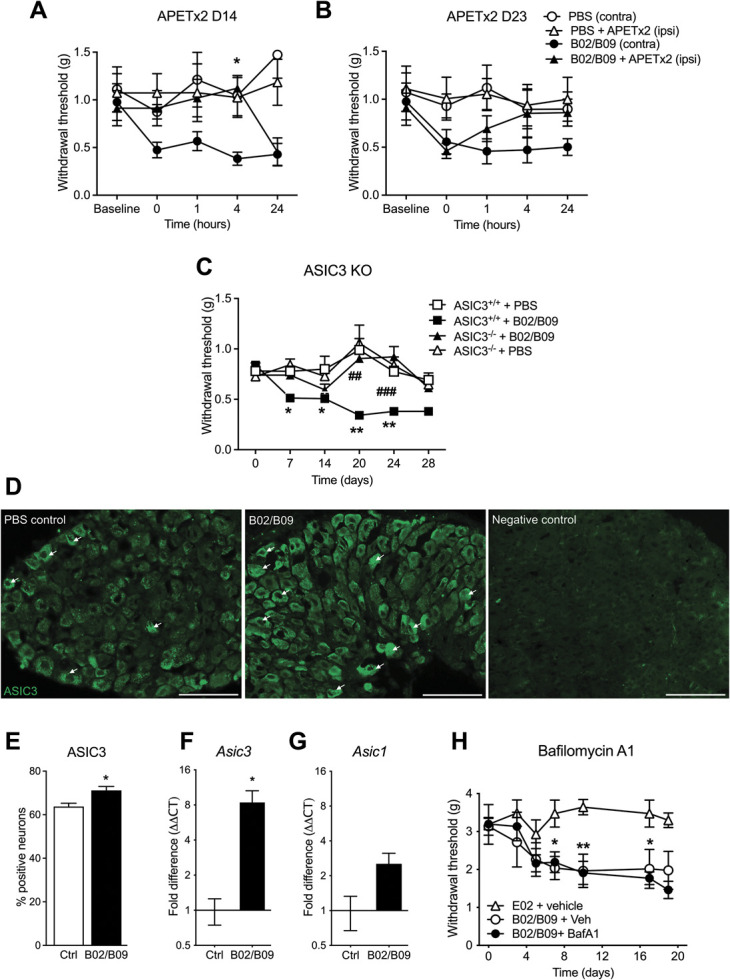
B02/B09-induced mechanical hypersensitivity is dependent on ASIC3 activation. Intra-articular administration of APETx2 (20 μM) into the ankle joint (A) day 14 and (B) day 23 post-mAbs injection transiently reverses mechanical hypersensitivity in the injected paw of B02/B09 mice, compared with the contralateral paw (n = 6-8/group). (C) ASIC3 KO mice do not develop mechanical hypersensitivity after B02/B09 injection in comparison with WT mice (n = 26-29 mice/group until day 14 and n = 9-19/group until day 28). Statistical significance (two-way ANOVA) between ASIC3^−/−^+B02/B09 and ASIC3^+/+^+B02/B09 is marked by #, and difference between ASIC3^+/+^+B02/B09 and ASIC3^+/+^+PBS is marked by *. (D–E) Fourteen days post-B02/B09 injection, there is a significant increase in ASIC3-positive neurons in L3-5 DRG, compared with the PBS-treated group (71.18 ± 0.8% vs 63.74 ± 0.77%). Negative control was stained only with the secondary antibody, scale bar = 100 µm. Arrows point to representative cells with positive ASIC3 staining. (F) *Asic3*, but not (G) *Asic1,* mRNA levels are significantly elevated in ankle joints of B02/B09-injected mice 14 days post-mAb injection compared with the PBS control group. (H) Repeated injections of bafilomycin A1 (25 μg/kg, s.c.) on days 12 to 16 post-B02/B09 administration did not reverse mechanical hypersensitivity induced by human B02/B09 compared with the vehicle-injected control group (0.01% DMSO in PBS). Data presented as mean±SEM, *P < 0.05, **or ## *P* < 0.01, ###*P* < 0.001. ANOVA, analysis of variance; ASIC3, acid-sensing ion channel 3; BafA1, bafilomycin A1; Ctrl, control; DRG, dorsal root ganglion.

As part of the bone resorbing process osteoclasts release protons into an extracellular compartment formed between osteoclasts and bone surface. As nociceptors express acid-sensing proton-gated ion channels such as ASIC3, acidification of the local bone environment can be detected by primary sensory neurons.^42,72^ To assess whether ASIC3 is involved in B02/B09-induced mechanical hypersensitivity, we treated mice with APETx2, a selective ASIC3 inhibitor, on 2 separate occasions. Intra-articular administration of APETx2 (20 µmol) reversed the mechanical withdrawal threshold in the ipsilateral paw of the injected ankle, both on day 14 (Fig. 6A) and day 23 post-B02/B09 administration (Fig. 6B). The effect was detectable 1 hour postinjection at day 14 and lasted up to 24 hours after administration when tested on day 23, compared with the B02/B09 contralateral paw of the saline-injected ankle in both experiments. Intra-articular administration of APETx2 or saline did not change the mechanical paw threshold in the PBS-treated group.

To further strengthen the observations that B02/B09-induced mechanical hypersensitivity is ASIC3-dependent, we used ASIC3-deficient mice. ASIC3 WT animals developed significant mechanical allodynia from day 7 up to day 28 after B02/B09 administration (Fig. 6C) compared with the ASIC3 WT PBS-treated group. In contrast, ASIC3-deficient mice did not exhibit any significant mechanical allodynia after B02/B09 administration compared with the ASIC3 KO PBS-treated group. Collectively, these results support the involvement of ASIC3 in pain-related behaviors induced by B02/B09 administration.

To assess whether increased osteoclast activity induced changes in ASIC3 expression in primary sensory neurons, we performed ASIC3 immunohistochemistry on L3-L5 DRG from B02/B09-injected mice and PBS controls. Fourteen days post injection, the percentage of ASIC3-positive neurons was significantly increased in the B02/B09-treated group (Figs. 6D and E) compared with the PBS treated group (71.18 ± 0.8% vs 63.74 ± 0.77%). Because ASIC channels are also expressed by osteoclasts and involved in differentiation and acidification, we also assessed gene expression of *Asic1* and *Asic3* in the ankle joints of B02/B09 mice, 14 days after antibody injection. Interestingly, only *Asic3* mRNA expression (Fig. 6F) was significantly increased compared with the PBS group while *Asic1* mRNA expression (Fig. 6G) remained unchanged.

Because ASIC3 can be activated by protons, we investigated whether blocking proton release through vacuolar-H^+^-ATPase from osteoclasts attenuates mechanical hypersensitivity induced by B02/B09. Repeated dosing of bafilomycin A1 (25 µg/kg), a potent and specific inhibitor of V-ATPase that prevents the acidification of vesicles present in the osteoclast ruffled membrane, on days 12 to 16 post-B02/B09 injection did not alter mechanical hypersensitivity in the B02/B09 group (Fig. 6H). This suggests that B02/B09-induced pain-like behavior is not directly linked to acidification but that other osteoclast-derived ligands are involved in ASIC3 activation instead.

### 3.7. B02/B09-induced mechanical hypersensitivity is accompanied by increased levels of lysophosphatidylcholine and increased secretory phospholipase A2 activity

**Figure 7.**
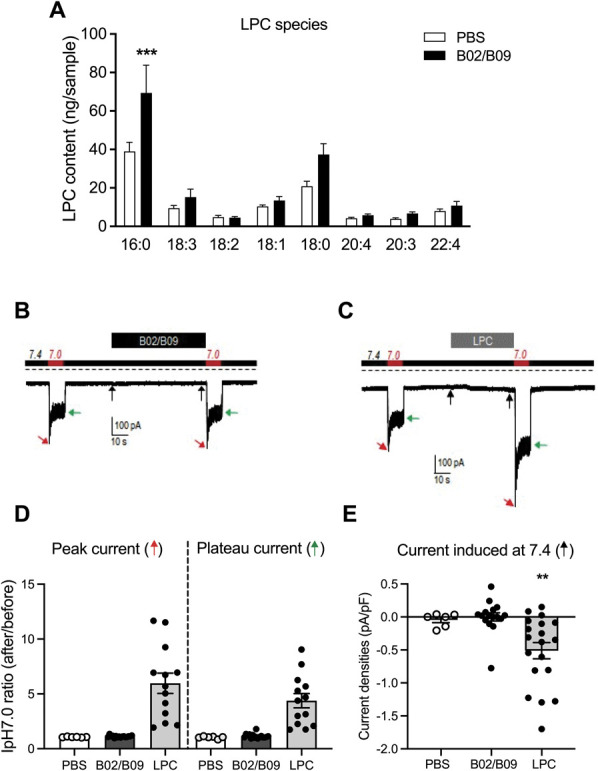
B02/B09-induced mechanical hypersensitivity is associated with increased levels of LPC 16:0. (A) Mass spectrometry analysis of total LPC concentration reveals that LPC 16:0 is the only LPC species significantly increased in bone marrow samples from B02/B09-injected mice, compared with PBS control (69.4 ± 12.5 ng/sample vs 39.0± 4.0 ng/sample, n = 6-7 mice/group, ***P < 0.001). (B–C) Typical ASIC3 currents recorded in patch clamp (HP -80 mV) from HEK293-transfected cells. Currents were elicited by extracellular pH drops from resting pH 7.4 to pH 7.0, before and after acute exposure of the cells to (B) B02/B09 (1 µg/mL each for 1 minute) or to (C) LPC 16:0 (10 µM for 25-50 seconds), as indicated by the timelines above current traces. Dotted lines indicate the zero-current level, and arrows show the effects of B02/B09 or LPC 16:0 on the basal current (black arrows), the pH 7.0-evoked peak current (red arrows), or the pH 7.0-evoked plateau current (green arrows). (D) Statistical analysis of the effects of B02/B09 and its vehicle (PBS) or LPC 16:0 on pH 7.0-evoked ASIC3 current. Data are presented as the peak and plateau current ratios after/before applications of B02/B09 and its vehicle (PBS) or LPC 16:0 (n = 6-16), ***P < 0.001. (E) Effects of B02/B09, its vehicle (PBS) or LPC 16:0 on the current densities recorded at resting pH 7.4 from ASIC3-expressing HEK cells (n = 6-19, **P < 0.01). ASIC3, acid-sensing ion channel 3; HEK, human embryonic kidney; LPC, lysophosphatidylcholine.

Small extracellular pH variations are considered the dominant, endogenous signals that trigger ASIC3 activation. However, certain lipids, such as LPC, induce constitutive activation of ASIC3 at a physiological pH of 7.4 without any extracellular acidification. Furthermore, LPC is increased in joint exudates of patients with RA.^40,58^ Therefore, we sought to investigate whether B02/B09-induced pain-like behavior is accompanied by increased LPC levels that could sensitize ASIC3. We assessed the levels of different LPC species in bone marrow extracts of B02/B09-treated mice and controls using mass spectrometry. Of 8 detected LPC species, we found that LPC 16:0 was the most abundant (Fig. 7A) and significantly increased in the B02/B09 group (69.38 ± 12.5 ng/sample) compared with control (38.96 ± 4.03 ng/sample).

To ascertain whether B02/B09 could directly activate and/or potentiate ASIC3 currents, we undertook patch-clamp experiments in HEK293 cells transfected with ASIC3. As expected, a typical ASIC3 current was recorded after an extracellular pH drop from pH 7.4 to pH 7.0, with a transient peak and a sustained plateau (Figs. 7B and C). No significant changes of this pH 7.0-evoked ASIC3 current was observed after acute exposure of the cells to B02/B09 (1 µg/mL for each) compared with its vehicle (Fig. 7D). B02/B09 was also unable to activate any ASIC3 current at resting pH 7.4 (Fig. 7E). By contrast, and as previously shown,^58^ LPC 16:0 extracellular application both activated ASIC3 at resting pH 7.4 and potentiated its pH 7.0-evoked current (Figs. 7C–E). Together, these data demonstrated that, contrary to LPC, the B02/B09 mix had no acute effects on ASIC3 channel activity.

### 3.8. B02/B09-induced pain-like behavior and bone erosion are associated with increased secretory phospholipase A2 activity

**Figure 8.**
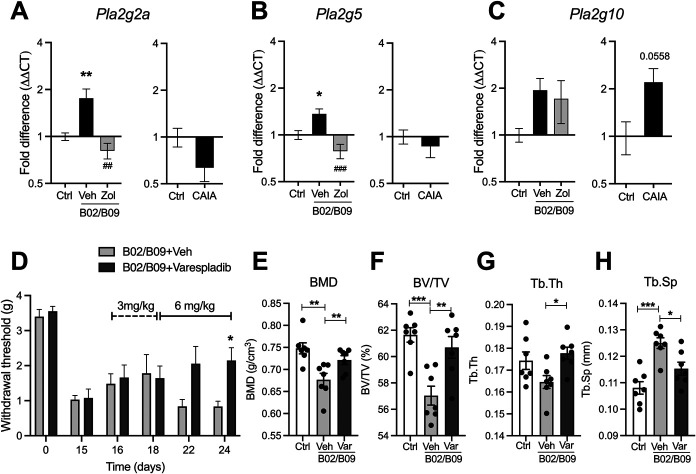
B02/B09-induced pain-like behavior and bone erosion are associated with increased sPLA_2_ activity. (A) *Pla2g2a* and (B) *Pla2g5*, but not (C) *Pla2g10,* mRNA levels are significantly elevated in the ankle joints of B02/B09-injected mice compared with PBS-injected mice. Treatment with zoledronate prevents the increase in gene expression of *Pla2g2a* and *Pla2g5* but not *Pla2g10* (n = 6 mice/group). (A) *Pla2g2a*, (B) *Pla2g5*, and (C) *Pla2g10* mRNA levels are not elevated in the ankle joints from mice subjected to CAIA collected during the inflammatory phase (day 16), compared with joints from control mice (n = 5/group). mRNA data were normalized to *Rplp2* mRNA levels and are presented as fold changes. Statistical significance (1-way ANOVA) between B02/B09 and control is marked by *, and difference between B02/B09 and B02/B09+Zol is marked by #. (D) An escalation-dose study with varespladib (3 mg/kg on days 16-18 and 6 mg/kg on days 19-24 post-B02/B09 administration) attenuates pain-like behavior in mice injected with human B02/B09 compared with B02/B09 mice treated with vehicle (20% DMSO in PBS) (n = 6-7/group). The dashed line indicates dosing period with 3 mg/kg varespladib and the solid line indicates the dosing period with 6 mg/kg varespladib. Statistical significance (2-way ANOVA) between B02/B09 group treated with varespladib and B02/B09 group treated with vehicle is marked by *. Micro-CT analysis of talus bone shows that varespladib significantly reverses B02/B09-induced bone erosion increasing (E) BMD, (F) BV/TV, and (G) Tb.Th and decreasing (H) Tb.Sp. compared with B02/B09 mice treated with the vehicle (n = 7/group). Data presented as mean±SEM, *, *P* < 0.05, **, ##*P* < 0.01, ***, ###*P* < 0.001. ANOVA, analysis of variance; BMD, bone mineral density; CAIA, collagen antibody–induced arthritis; Ctrl, control; sPLA_2_, secretory phospholipase A2; Veh, vehicle, Var, varespladib; Zol, zoledronate.

Because B02/B09-injection leads to a significant elevation of LPC 16:0 content in the bone marrow, we assessed whether gene expression levels of sPLA_2_ isoforms involved in LPC production (*Pla2g2a, Pla2g5, and Pla2g10*) were increased in the ankle joints of B02/B09-injected mice. Fourteen days post antibody administration, *Pla2g2a* (Fig. 8A) and *Pla2g5* (Fig. 8B) mRNA levels in the B02/B09 group were significantly increased and, importantly, reduced in ankle joints of mice treated with zoledronate. *Pla2g10* mRNA levels in the B02/B09 groups receiving vehicle or zoledronate were unchanged compared with control (Fig. 8C). By contrast, *Pla2g2a* and *Pla2g5* expression in joints from the inflammatory phase of CAIA did not change and even showed a tendency towards being downregulated, although not statistically significant (Figs. 8A and B). *Pla2g10* was also unchanged in CAIA mice (Fig. 8C). Taken together, this suggests that the altered *Pla2g2a* and *Pla2g5* gene expressions in the B02/B09 group are unlikely to be associated with ongoing inflammation.

**Figure 9.**
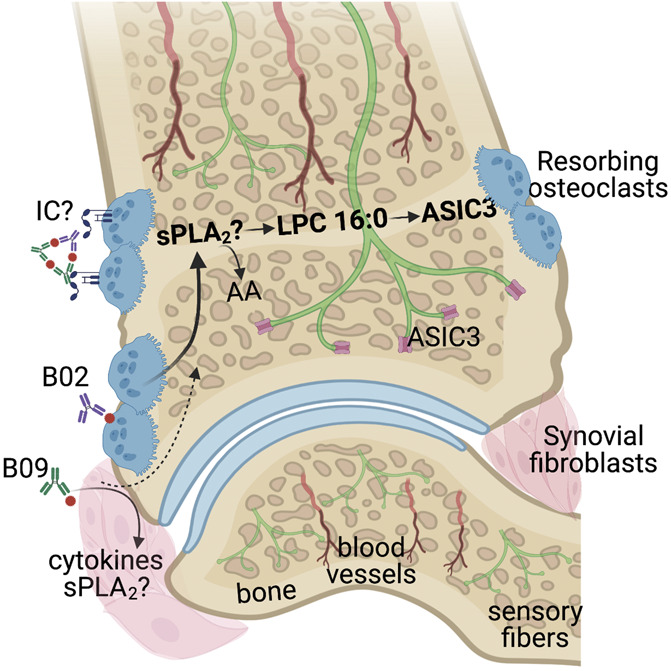
Proposed mechanism for B02/B09-induced bone erosion and mechanical hypersensitivity in the mouse joint. B02 and B09 mAbs bind their epitopes on osteoclasts and fibroblasts and/or form immune complexes (ICs) that stimulate Fcγ receptors on osteoclasts and drive osteoclastogenesis. As a result of B02 and B09 binding, several pronociceptive factors are released including sPLA_2_ which catalyzes the formation of LPC 16:0 that further activates ASIC3 located on the sensory nerve endings innervating the bone, inducing mechanical hypersensitivity. The exact cellular source of sPLA_2_ remains to be elucidated. ASIC3, acid-sensing ion channel 3; LPC, lysophosphatidylcholine; mAb, monoclonal antibody; sPLA_2_, secretory phospholipase A2.

Finally, to address whether B02/B09 mAb-induced pain-like behavior is dependent on elevated sPLA_2_ activity, mice with B02/B09-induced hypersensitivity were treated with varespladib, an inhibitor of the IIa, V, and X isoforms of secretory PLA_2_. A dose escalation study was performed, where 3 mg/kg/d was administrated once a day for 3 days (initiated day 16 post-B02/B09 injection), followed by 6 mg/kg twice a day for the next 5 consecutive days of the study. Repeated injections of varespladib resulted in significant reversal of mechanical hypersensitivity in the treated group at day 24 post-B02B09 injection, compared with mice that received vehicle (Fig. 8D). Importantly, treatment with varespladib significantly reversed B02/B09-induced bone erosion in the talus bone (Figs. 8E–H), suggesting that sPLA_2_ contributes to autoantibody-driven bone loss in this model (Fig. 9).

Lysophosphatidylcholine may serve as a substrate for autotaxin, the LPC to LPA converting enzyme that has been linked to nociception through the activation of LPA receptors (*Lpa1-6*).^38,95^ To rule out the possible LPA involvement in this model, we assessed the expression of autotaxin (*Enpp2*) and LPA receptors (*Lpa1-6*) in the ankle joints from mice injected with B02/B09 (Fig. S1A-E, available as supplemental digital content at http://links.lww.com/PAIN/B542); however, neither mRNA levels were elevated in the B02/B09 joints compared with control mice.

## 4. Discussion

In this study, we have linked bone erosion induced by 2 mAbs originally isolated from B cells of patients with RA to mechanical hypersensitivity in mice. The absence of edema and synovitis, lack of analgesic effect of naproxen, and modest change in inflammatory mediators suggest that B02/B09-induced pain-like behavior is independent of classical inflammatory processes. By contrast, we found that inhibiting osteoclast activity and ASIC3 signaling prevented the development of B02/B09-mediated pain-related behavior. Our data further suggest that sPLA_2_ and LPC 16:0 contribute to sensitization, representing a potential novel link between increased osteoclast activity and ASIC3-mediated hypersensitivity.

Rheumatoid arthritis has a preclinical phase before diagnosis, where levels of circulating autoantibodies are increasing but clinical signs of disease are absent or sparse.^84^ Autoantibodies, such as B09, that are reactive to antigens with different posttranslational modifications, such as citrullination, acetylation, and carbamylation, are detected during this period.^8,26,43^ In vitro studies have shown that B09 lacks stimulatory effects on osteoclasts^86^ but does bind nuclear antigens in activated neutrophils^55^ and stimulates migration of stressed fibroblast-like synoviocytes.^89^ Here, we show that after systemic administration to mice, B09 induces transient mechanical hypersensitivity in the absence of bone loss. The pronociceptive and osteoclast-activating actions of B02 are in line with our previous work.^50,98^ However, at that time, we ascribed the actions of B02 to binding of citrullinated epitopes; extensive analysis with new optimized assays has demonstrated that it does not have any detectable citrulline specificity^27,81^ and its antigen reactivity remains unknown. A potential caveat of our animal model is that the human-derived antibodies were developed against human posttranslational modifications and the exact same epitopes may not occur in mice. Although the lack of known B02 antigenicity, together with the use of only 2 patient-derived mAbs is a weakness of the study, we considered this RA synovial antibody combination to be a useful tool to study the pathogenic link between bone loss and pain in the absence of pronounced tissue inflammation.

Subclinical joint inflammation^9^ and the action of autoantibodies in a monomeric or immune complex form^33,47^ have been suggested to be sufficient to trigger osteoclast differentiation and bone erosion. Interestingly, in our model, we did not detect significant signs of synovitis or visible joint inflammation. Increased serum levels of TNF and IL-6 have been reported in the preclinical phase of RA^87^ and together can stimulate osteoclastogenesis directly^69^ and indirectly by upregulating RANKL in synovial fibroblasts.^34,93^ Although we did not assess systemic cytokine levels, there was only a modest upregulation of *Tnf*, *Il-6*, and *Ptgs2* in the joint. Of note, morphine reversed the hypersensitivity in B02/B09 mice but naproxen was without effect, indicating a prostaglandin-independent mechanism. We have not assessed other pain modalities in B02/B09 mice, and although we predict that these analgesics would mount a similar effect in other behavioral readouts, future studies assessing spontaneous behavioral changes are important. Taken together, pronounced “classical” inflammatory processes are unlikely to drive B02/B09-induced mechanical hypersensitivity indicating that other mechanisms, including osteoclast activity, have a more prominent role.

Accumulating data suggest that bone erosion does not only correlate with disease activity and synovial inflammation^56,101^ but may also occur before onset of joint inflammation.^47^ Considering this, we were intrigued that the B02 and B09 combination induced pain-related behaviors and bone erosion in the absence of synovitis. Interestingly, significant in vivo bone erosion in mice was detected only with the B02/B09 combination suggesting that these 2 antibodies initiate a combination of processes that are needed to significantly alter bone morphology. Consequently, 2 osteoclast inhibitors, zoledronate and T06,^71^ completely prevented the development of B02/B09-induced mechanical hypersensitivity. A potential weakness of our pharmacology studies is that zoledronate also alters microglia and macrophage activity, which can reduce pain.^77^ However, our findings are likely independent of these cells because the role of microglia in nociception is coupled to male mice^85^ and female mice were used in our study. Zoledronate's actions on macrophages in vivo are linked to cytokine release^45^; however, in our model, it did not reduce expression of the upregulated cytokines (*Tnf*, *Il-6*, or *Ptgs2*) in the joint. Although we cannot exclude that off-target effects may contribute to our observations, 2 osteoclast inhibitors with different mechanisms of action yielded similar antinociceptive results, which supports previous findings showing efficacy of osteoclast inhibitors in models of several difficult-to-treat pain states.^94^

Increased resorptive activity of osteoclasts is associated with acidification of the extracellular compartment.^80^ Acid-sensing ion channel 3, expressed by peripheral nociceptors, becomes activated at pH ≈ 7.2 and is likely important in the nonadapting pain caused by tissue acidosis, particularly because it has the ability to sensitize nociceptors to other types of stimuli.^18^ As preclinical evidence suggests a key role of ASIC3 in inflammatory^36,65,100^ and noninflammatory pain with bone pathology, such as osteoarthritis^39^ and osteoporosis,^20,31^ we examined whether B02/B09-induced mechanical hypersensitivity was dependent on ASIC3 activation. Using APETx2, an inhibitor of ASIC3-containing channels, as well as a global, functional ASIC3 knockout, we identified ASIC3 signaling as a critical component of B02/B09-induced hypersensitivity. A potential caveat of the pharmacological approach is that APETx2 was demonstrated to block Na_v_1.8 currents^6^; however, by complementing it with a genetic approach, we have excluded the potential off-target involvement. We also saw an upregulation of ASIC3 protein in DRG neurons 2 weeks after injection of B02/B09, suggesting either a feed-forward regulation and/or induction through subclinical inflammation, as suggested previously.^66,73,97,103^ To investigate whether resorbing osteoclasts contribute to B02/B09-induced hypersensitivity through the release of protons, we used bafilomycin A1, an inhibitor of V-ATPase, which is the primary cellular mechanism responsible for osteoclast acidification.^80^ Surprisingly, although bafilomycin A1 attenuates osteoclast-mediated pain-related behaviors in cancer models,^35,66^ it did not diminish B02/B09-induced mechanical hypersensitivity in mice. We speculate that alternative osteoclast-derived ligands couple osteoclast activity with ASIC3 sensitization and protons are not required for induction of B02/B09-induced hypersensitivity.

Importantly, ASIC3 acid-induced currents are potentiated by lipids such as LPC, which can also^18,58^ directly activate ASIC3, even in the absence of extracellular acidification.^58^ Intraplantar injection of LPC 18:1^78^ and LPC 16:0^28^ induces mechanical and cold hypersensitivity, respectively. Although the exact mechanisms behind LPC-induced pain-like behavior are still under investigation, some studies point towards ASIC3 as the main mediator of LPC-induced hypersensitivity.^37,58^ Of note, elevated levels of LPC and sPLA_2_, an enzyme family needed for LPC production, have been reported in plasma and synovial fluid of patients with RA and osteoarthritis and individuals with painful joints,^30,49,58,102^ suggesting that LPC may also contribute to pain in humans. Furthermore, injection of sPLA_2_ induces hypersensitivity,^12,13^ and several studies indicate a role of sPLA_2_ in several different models of persistent pain.^46,74,90,92^ Of note, we found (1) elevated levels of LPC 16:0 in bone marrow, (2) direct activation and potentiation of ASIC3 by LPC 16:0 and not B02/B09 in vitro, (3) increased mRNA levels for 2 sPLA_2_ isoforms (*Pla2g2* and *Pla2g5*) in the ankle joints of B02/B09-injected mice, and (4) that inhibition of sPLA_2_ reversed both mechanical hypersensitivity and bone erosion induced by B02/B09 mAbs. Thus, our data point to a role of sPLA_2_ and LPC in pain behavior in our model.

We have not yet determined the cellular source of sPLA_2_ that contributes to B02/B09-induced hypersensitivity, but intriguingly, in specific conditions, osteoclasts, chondrocytes, and fibroblasts-like synoviocytes can generate sPLA.^2,10,53,61,76^ Thus, we speculate that either B02-activated osteoclasts or fibroblasts, activated by B09, release sPLA_2_ followed by the subsequent formation of extracellular LPC, particularly LPC 16:0, that ultimately activates ASIC3 on nociceptors and osteoclasts. Because LPC 16:0, unlike acid, activates ASIC3 in a noninactivating manner it is likely that it will sensitize primary afferent neurons to mechanical stimuli.^18,79,96^ Alternatively, LPC 16:0 can directly sensitize ASIC3 to mechanical stimuli, leading to alterations of mechanical neuronal response of primary afferent neurons. Finally, LPC could be converted to LPA and act through LPA receptors present on nociceptors and osteoclasts, although we did not find any indications of increased expression of the LPC to LPA converting enzyme autotaxin or LPA receptors in the B02/B09 joints. Furthermore, low levels of cytokines released from joint cells and changes in pH in response to B02/B09, together with the presence of sPLA_2_-mediated products, may sensitize the nociceptors.

In summary, our work presents a novel mechanism on how RA autoantibodies can drive mechanical hypersensitivity. Our findings couple bone erosion to pain during stages of subclinical inflammatory activity and thus offer a step forward in understanding the mechanisms of arthralgia in individuals with “preclinical RA”. This is important because although the inflammatory component of joint pain has been extensively studied in arthritis, we still lack understanding on how pain is induced and sustained in the early phase of arthritis, when joint swelling cannot be identified through physical examination, but bone erosion and pain are already present. Pinpointing the interactions between activated osteoclasts and sensory nerve fibers innervating the joints will allow for better treatment of arthralgia but also other pain conditions characterized by altered bone metabolism.

## Conflict of interest statement

The authors have no conflicts of interest to declare.

## Appendix A. Supplemental digital content

Supplemental digital content associated with this article can be found online at http://links.lww.com/PAIN/B542.

## Supplemental video content

A video abstract associated with this article can be found at http://links.lww.com/PAIN/B543.

## Supplementary Material

SUPPLEMENTARY MATERIAL
