# Supplementary material for: Antibody-induced pain-like behavior and bone erosion: links to subclinical inflammation, osteoclast activity, and acid-sensing ion channel 3–dependent sensitization
Source: Pain. 2021 Nov 19;163(8):1542–59. doi: 10.1097/j.pain.0000000000002543 (PMC9341234; doi:10.1097/j.pain.0000000000002543)
Supplement: SUPPLEMENTARY MATERIAL [file jop-163-1542-s001.pdf]

Supplementary figures

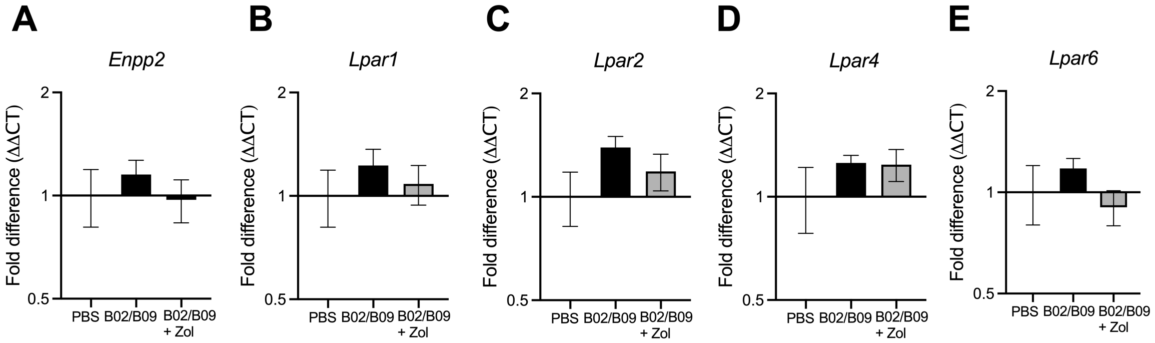

Fig S1. B02/B09-induced mechanical hypersensitivity is not accompanied by increased expression of autotaxin or LPA receptors in the ankle joint. mRNA levels of **(A)** Enpp2, **(B)** Lpar1, **(C)** Lpar2, **(D)** Lpar4 and **(E)** Lpar6 are not elevated in joints harvested from B02/B09, compared to PBS-injected mice, and are not altered by zoledronate treatment. Lpar3 and Lpar5 Ct values are below the linear range of detection (not shown in the figure). mRNA data were normalized to Rplp2 mRNA levels and are presented as fold change, (n=5-6/group). Zol, zoledronate.

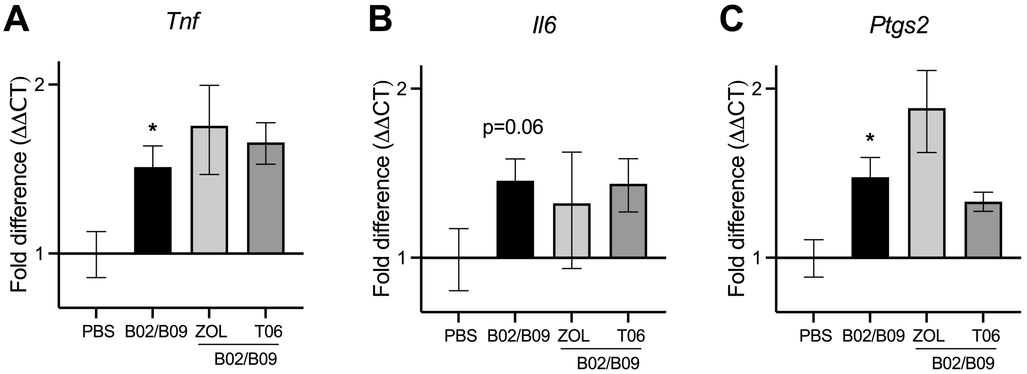

Fig S2. Treatment with osteoclast inhibitors does not decrease the expression of Tnf, Il-6 and Ptgs2 in joints from B02/B09-injected mice. (A) Tnf, **(B)** Il-6 and **(C)** Ptgs2 mRNA levels are elevated in the joints from B02/B09-injected mice, compared to PBS control, however they are not significantly altered by two osteoclast inhibitors: zoledronate and T06.

mRNA data were normalized to Rplp2 mRNA levels and are presented as fold change, (n=6-14/group), statistical significance (one-way ANOVA) between B02/B09 and PBS-injected control is marked by *. Zol, zoledronate, T06 Tanshinone IIA sulfonic sodium.
